# Clinicopathological factors influencing the outcomes of surgical treatment in patients with T4a hypopharyngeal cancer

**DOI:** 10.1186/s12885-017-3880-6

**Published:** 2017-12-29

**Authors:** Sang-Yeon Kim, Young-Soo Rho, Eun-Chang Choi, Min-Sik Kim, Joo-Hyun Woo, Dong Hoon Lee, Eun Jae Chung, Min Woo Park, Da-Hee Kim, Young-Hoon Joo

**Affiliations:** 1 Department of Otolaryngology-Head and Neck Surgery, College of Medicine, The Catholic University of Korea, Seoul, Republic of Korea; 2 Department of Otorhinolaryngology-Head and Neck Surgery, Ilsong Memorial Institute of Head and Neck Cancer, Hallym University, College of Medicine, Seoul, Republic of Korea; 3 Department of Otorhinolaryngology, Yonsei University, College of Medicine, Seoul, Republic of Korea; 4 Department of Otolaryngology Head and Neck Surgery, Gachon University Gil Hospital, Incheon, Korea; 5 Department of Otolaryngology-Head and Neck Surgery, Chonnam National University Medical School & Chonnam National University Hwasun Hospital, Hwasun, Korea; 6 Department of Otorhinolaryngology–Head and Neck Surgery, Seoul National University College of Medicine, Seoul, Korea; 7 Department of Otolaryngology, Head and Neck Surgery, Bucheon St. Mary’s Hospital, College of Medicine, The Catholic University of Korea, 2 Sosa-dong, Wonmi-gu, Bucheon, Kyounggi-do 420-717 Republic of Korea

**Keywords:** Head and neck neoplasms, Hypopharynx, Squamous cell carcinoma, Surgery, Treatment outcome

## Abstract

**Background:**

The purpose of this study was to determine prognostic factors influencing outcomes of surgical treatment in patients with T4a hypopharyngeal cancer.

**Methods:**

The present study enrolled 93 patients diagnosed with T4a hypopharyngeal cancer who underwent primary surgery between January 2005 and December 2015 at six medical centers in Korea. Primary tumor sites included pyriform sinus in 71 patients, posterior pharyngeal wall in 14 patients, and postcricoid region in 8 patients. Seventy-two patients received postoperative radio(chemo)therapy.

**Results:**

Five-year disease-free survival (DFS) and disease-specific survival (DSS) rates were 38% and 45%, respectively. In univariate analysis, 5-year DFS was found to have significant and positive correlations with margin involvement (*p* < 0.001) and extracapsular spread (*p* = 0.025). Multivariate analysis confirmed that margin involvement (hazard ratio (HR): 2.81; 95% confidence interval (CI): 1.49-5.30; *p* = 0.001) and extracapsular spread (HR: 2.08; 95% CI: 1.08-3.99; *p* = 0.028) were significant factors associated with 5-year DFS. In univariate analysis, cervical lymph node metastasis (*p* = 0.048), lymphovascular invasion (*p* = 0.041), extracapsular spread (*p* = 0.015), and esophageal invasion (*p* = 0.033) were significant factors associated with 5-year DSS. In multivariate analysis, extracapsular spread (HR: 2.98; 95% CI: 1.39-6.42; *p* = 0.005) and esophageal invasion (HR: 2.87; 95% CI: 1.38-5.98; *p* = 0.005) remained significant factors associated with 5-year DSS.

**Conclusion:**

Margin involvement and extracapsular spread are factors influencing recurrence while extracapsular spread and esophageal invasion are factors affecting survival in patients with T4a hypopharyngeal cancer treated by primary surgery.

## Background

Hypopharyngeal cancer represents approximately 7% of all cancers of the upper aerodigestive tract. More than 95% of these cancers are squamous cell carcinomas [1]. Among head and neck cancers, hypopharyngeal squamous cell carcinoma (HPSCC) is known to have the worst prognosis. In one literature, 5-year survival rates for stage III and IV HPSCC have been reported to be 36% and 24%, respectively [2]. A relatively poor prognosis and frequently advanced stage at diagnosis are due to the relative lack of symptoms for early-stage of this disease at this region.

Treatment for HPSCC remains controversial. Some authors advocate for the use of primary radiotherapy alone or in combination with chemotherapy for HPSCC [3–6]. However, treatment of T4a HPSCC continues to fuel debate. Because HPSCC is a relatively rare disease, optimal initial treatment for T4a HPSCC has not been evaluated in any large, prospective, randomized study. Patients exhibiting cartilage invasion have poorer survival outcomes after irradiation. Therefore, T4a HPSCC with thyroid cartilage invasion is considered a distinct subcategory [7]. Clinical practice guidelines recommend upfront hypopharyngectomy with adjuvant radiotherapy for T4a HPSCC because rates of successful salvage surgery after failure of nonsurgical treatment are low [8]. The objective of this study was to present treatment results of primary surgery and identify possible prognostic factors affecting treatment outcomes in patients with T4a HPSCC.

## Methods

Patients with pathologically confirmed HPSCC were recruited from six general hospitals for this multicenter study organized by a research committee of the Korea Society of Thyroid Head and Neck Surgery. Data for the following clinicopathological parameters in patients with T4a HPSCC who underwent primary surgery between 2005 and 2015 were collected: age, gender, comorbidities, tumor site and stage, postoperative treatment, pathologic specimen analysis, tumor recurrence, death, and cause of death. Tumor stage was determined based on the 2009 American Joint Committee on Cancer TNM classification. Data for a total of 416 patients with T4a HPSCC who underwent primary surgery over the 11-year period (2005 to 2015) were collected from the six centers. Among these patients, 323 were excluded because they received chemoradiotherapy for primary treatment or had recurrence of the primary tumor. Finally, a total of 93 patients were included in the study. Their mean follow-up period was 26.1 months (range, 1–118 months). Those who had positive or close margins and those with advanced T stage, lymphovascular invasion, perineural invasion, multiple nodal metastases, or extracapsular spread received additional treatment.

### Statistical analysis

Survival was determined using the Kaplan-Meier method. Relationships between categorical variables were analyzed by Fisher’s exact test or Chi-square test. A *p*-value of less than 0.05 was considered statistically significant. All calculations were performed using SPSS software ver. 16.0 (SPSS, Chicago, IL, USA). Disease-free survival (DFS) was defined as the time from the date of commencement of treatment to tumor recurrence. Disease-specific survival (DSS) was defined as the time from the first day of treatment to the date of death from hypopharyngeal cancer.

## Results

### Patient demographics

The male to female ratio was 86:7. The median age of all patients was 63.5 years (range, 34–84 years). Primary tumor sites included pyriform sinus in 71 patients, posterior pharyngeal wall in 14 patients, and postcricoid region in 8 patients. Regarding pathologic disease stage of cervical lymph nodes, 12, 8, 2, 41, 25, and 5 patients were found to have stage N0, N1, N2a, N2b, N2c, and N3, respectively. Detailed patient characteristics are summarized in Table 1.

**Table 1:** Demographic profiles of patients with T4a hypopharyngeal squamous cell carcinoma (*n* = 93)

Parameter	No of patients (%)
Age (years)	
≤ 60	65 (69.9)
> 60	28 (30.1)
Gender	
Male	86 (92.5)
Female	7 (7.5)
Primary tumor site	
Pyriform sinus	71 (76.3)
Posterior pharyngeal wall	14 (15.1)
Postcricoid region	8 (8.6)
N classification	
N0	12 (12.9)
N1	8 (8.6)
N2a	2 (2.2)
N2b	41 (44.1)
N2c	25 (26.9)
N3	5 (5.4)
Adjuvant therapy	
Radiation only	33 (35.5)
Concurrent chemoradiation	39 (41.9)
None	21 (22.6)
Margin involvement	
Yes	27 (29.0)
No	66 (71.0)
Histologic differentiation	
Well differentiated	18 (19.4)
Moderately differentiated	56 (60.2)
Poorly differentiated	11 (11.8)
Unknown	8 (8.6)
Lymphovascular invasion	
Yes	56 (60.2)
No	30 (32.3)
Unknown	7 (7.5)
Extracapsular spread	
Yes	46 (49.5)
No	40 (43.0)
Unknown	7 (7.5)

Regarding surgery types, total laryngectomy with partial pharyngectomy was performed in 41 patients, while partial laryngectomy with partial pharyngectomy was performed in 18 patients. Total laryngopharyngectomy with cervical esophagectomy was performed in 12 patients. Total laryngopharyngectomy was performed in 11 patients. Total laryngopharyngoesophagectomy was also performed in 11 patients (Table 2). For reconstruction of hypopharyngeal defects, radial forearm free flap was performed in 34 patients, anterolateral thigh free flap was performed in 11 patients, gastric pull-up was performed in 11 patients, pectoralis major myocutaneous flap was performed in 10 patients, and jejunal free flap was performed in 7 patients. Three kinds of adjuvant chemotherapy regimens were used for these patients: cisplatin, cisplatin plus 5-fluorouracil, and cetuximab. Radiation dose ranged from 4000 cGy to 6640 cGy, with a median dose of 6048 cGy.

**Table 2:** Primary surgery and reconstruction types

	No of patients (%)
Primary Surgery	
Partial laryngectomy with partial pharyngectomy	18 (19.4)
Total laryngectomy with partial pharyngectomy	41 (44.1)
Total laryngopharyngectomy	11 (11.8)
Total laryngopharyngectomy with cervical esophagectomy	12 (12.9)
Total laryngopharyngoesophagectomy	11 (11.8)
Reconstruction	
Radial forearm free flap	34 (36.6)
Anterolateral thigh free flap	11 (11.8)
Pectoralis major myocutaneous flap	10 (10.8)
Gastric pull-up	11 (11.8)
Jejunal free flap	7 (7.5)
Primary closure	20 (21.5)

### Disease-free survival

**Fig. 1:**
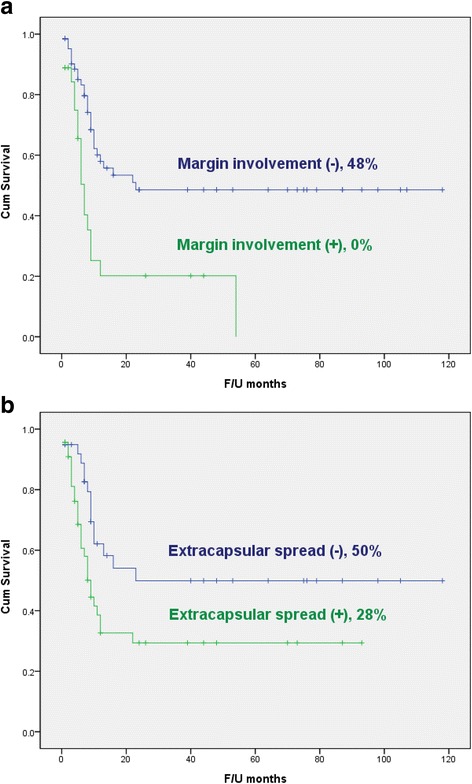
Kaplan-Meier disease-free survival curves according to resection margin involvement (**a**) and extracapsular spread (**b**). Resection margin involvement (*p* < 0.001) and extracapsular spread (*p* = 0.025) showed significant associations with 5-year disease-free survival

Recurrences or metastases occurred in 46 patients. Eighteen cases had distant metastasis while 14 cases had both regional recurrence and distant metastasis. Eleven cases had recurrence or metastasis in the neck. One case of recurrence or metastasis was found at the primary site. One case had both local and regional recurrences while one case had both local recurrence and distant metastasis. The recurrence rate was 49.5% (46/93) over a mean observation period of 26.1 months. Five-year DFS was 38%. Five-year survival rates for each contributing clinicopathologic factor analyzed are shown in Table 3. In univariate analysis, resection margin involvement (*p* < 0.001) and extracapsular spread (*p* = 0.025) were significant prognostic factors for DFS (Fig. 1). In multivariate analysis, margin involvement (hazard ratio (HR): 2.81; 95% confidence interval (CI): 1.49-5.30; *p* = 0.001) and extracapsular spread (HR: 2.08; 95% CI: 1.08-3.99; *p* = 0.028) remained significant predictors for unfavorable 5-year DFS. Adjuvant (chemo)radiotherapy rate for patients with margin positive was 77.8% (21 out of 27 patients). It was 82.6% (38 out of 46 patients) for patients with extracapsular spread. However, there was no significant difference in DFS between the group receiving adjuvant (chemo)radiotherapy and those without receiving such therapy (*p* = 0.790 for patients with margin positive and *p* = 0.180 for patients with extracapsular spread).

**Table 3:** Log-Rank test for clinicopathological factors

Parameter	DFS (%)	*p* value	DSS (%)	*p* value
Age		0.437		0.216
≥ 60 yrs	38		41	
< 60 yrs	46		57	
Gender		0.437		0.520
Male	37		44	
Female	41		53	
Primary tumor site		0.148		0.554
Pyriform sinus	38		45	
Posterior pharyngeal wall	32		32	
Postcricoid region	50		62	
Cervical metastasis		0.301		0.048*
Yes	34		40	
No	57		78	
Adjuvant therapy		0.316		0.106
Radiation only	39		54	
Concurrent chemoradiation	34		34	
None	59		71	
Margin involvement		<0.001*		0.124
Yes	0		27	
No	48		53	
Histologic differentiation		0.399		0.244
Well differentiated	57		68	
Moderately differentiated	36		43	
Poorly differentiated	32		30	
Lymphovascular invasion		0.426		0.041*
Yes	35		34	
No	41		63	
Extracapsular spread		0.025*		0.015*
Yes	28		34	
No	50		61	
Esophageal invasion		0.197		0.033*
Yes	21		30	
No	43		52	

*DFS* Disease-free survival, *DSS* disease-specific survival

*Significant at *p* < 0.05

### Disease-specific survival

**Fig. 2:**
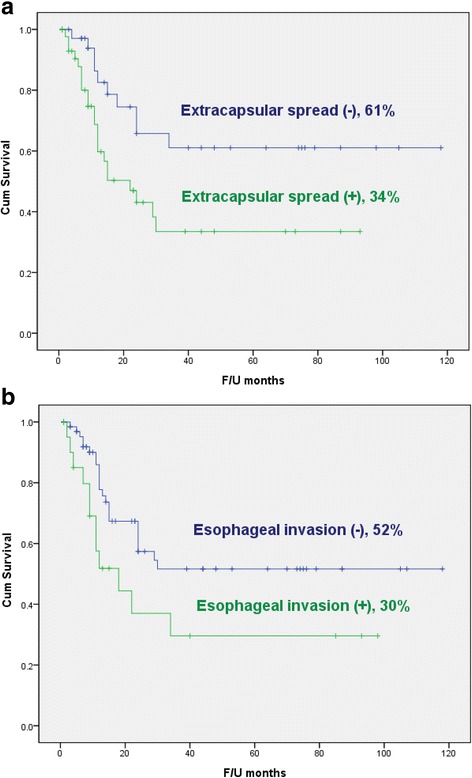
Kaplan-Meier 5-year disease-specific survival curves according to extracapsular spread (**a**) and esophageal invasion (**b**). Extracapsular spread (*p* = 0.015) and esophageal invasion (*p* = 0.033) showed significant associations with 5-year disease-specific survival

Five-year DSS for all patients who underwent primary surgery were 45%. Thirty-seven patients died, including 35 deaths from HPSCC and two deaths from other diseases. By univariate analysis, extracapsular spread (*p* = 0.015), esophageal invasion (*p* = 0.033), lymphovascular invasion (*p* = 0.041), and cervical lymph node metastasis (*p* = 0.048) showed significant positive correlations with 5-year DSS (Fig. 2). In multivariate analysis, extracapsular spread (HR: 2.98; 95% CI: 1.39-6.42; *p* = 0.005) and esophageal invasion (HR: 2.87; 95% CI: 1.38-5.98; *p* = 0.005) remained significant factors associated with 5-year DSS.

## Discussion

HPSCC is known to have poor prognosis among head and neck cancers. It is mostly found at advanced stage [9]. In the past, radical ablative surgery was conducted in hypopharyngeal cancer patients. It resulted in loss of speech and swallowing dysfunction. Total laryngectomy was introduced by Billroth et al. in 1873. It has been used as the main surgical choice for a few decades [3–5]. With development of surgical techniques, many types of conservation surgeries have enabled surgeons to restore the function of the larynx for patients. From 1990s, chemoradiotherapy has been widely used as an alternative option for radical surgery in HPSCC. Some authors have reported that advanced chemoradiotherapy technique can provide outcome equivalent to primary surgery, even in patients with advanced stage HPSCC [6, 7]. However, for patients with advanced stage HPSCC, oncologic outcomes of chemoradiotherapy are generally inferior to those of primary surgery [4–6]. Especially, patients with cartilage invasion have poor oncologic outcomes when they are treated with radiotherapy [7, 10]. Advanced-stage tumors with bone and cartilage invasion might harbor a hypoxic microenvironment that causes resistance to radiotherapy [11]. Recently, Scherl et al. have reported that prognosis of patients with advanced hypopharyngeal and laryngeal cancer after chemoradiotherapy is worse than that after primary surgery [12]. They concluded that proper selection of treatment modality could increase their survival rate. They also reported that 5-year DSS in the primary surgery group was significant higher than that in the chemoradiotherapy group which showed soft tissue invasion and cartilage invasion (5-year DSS: 51.1% in the primary surgery group vs. 28.5% in the chemoradiotherapy group, *p* < 0.05) [12].

In our series, extracapsular spread was significantly associated with rates of recurrence and survival on multivariate analysis. Many studies have reported that extracapsular spread is an indicator of poor prognosis of patients with HPSCC. Prim et al. have analyzed data of 128 patients with laryngeal and hypopharyngeal cancer and found that 3-year survival rate in patients without extracapsular spread is significantly higher than that in patients with extracapsular spread (73.4% vs. 28.9%, *p* < 0.001) [13]. Brasilino has analyzed data of 170 patients with laryngeal and hypopharyngeal cancer and reported that 5-year DFS of patients without cervical metastases is significantly higher than that in patients with macroscopic extracapsular spread (56.8% vs. 10.2%, *p* < 0.0001) [14]. In the aspect of distant metastasis, extracapsular spread has a negative effect on prognosis. According to Vaidya et al., in patients who underwent surgical resection, majority of them (18 out of 24 patients) showed recurrences for those who had cervical metastases with extracapsular nodal spread involving distant sites, especially to the lung [15].

Another significant indicator of recurrence in this study was margin status. It is known that inadequate resection can lead to increased likelihood of disease recurrence and poorer odds of survival for patients [16–18]. Ravasz has shown that locoregional recurrence observed in 20% of 80 head and neck cancer patients is correlated with tumor positive margins [18]. In our series, involved margins were found in 29% of cases. Five-year DFS of patients with negative margins was 48% and that of patients with positive margins was 0% (*p* < 0.001).

Esophageal invasion was identified as an another negative prognostic factor in our study. It is well-known that patients with advanced cancer simultaneously involving the hypopharynx and cervical esophagus have very poor prognosis. Five-year survival of these patients is approximately 20–30% [19]. Wang et al. have reported about survival and complication rates of patients who have cancer involvement of both hypopharynx and cervical esophagus [3]. They have explained the reason for such difference as follows: (1) Cervical esophagus has abundant lymphatics in the submucosa and the muscularis mucosa; (2) Cervical esophageal cancer is associated with a higher rate of mediastinal lymph node metastasis than hypopharyngeal cancer [20, 21]; and (3) Carcinoma of the cervical esophagus frequently invades into the posterior membranous portion of the trachea. These reasons and theories could be used to explain results of our study showing that HPSCC with esophageal invasion showed poor outcomes in terms of DSS.

This study has several limitations. First, the number of patients was relatively small. However, HPSCC is quite rare among head and neck cancers and most patients are diagnosed in very advanced stage. Therefore, data collection was the most difficult part of such study. This was why we used a multi-center study design initially. The second limitation of this study was its retrospective nature. Despite these limitations, our study provided an important guide for treatment of T4a HPSCC and suggested prognostic factors for outcomes of surgical treatment. Lastly, patients with HPSCC who were treated by different modalities were not included.

## Conclusions

The current study is the largest and the most robust analysis to identify specific prognostic factors in patients with T4a HPSCC treated by primary surgery. Margin involvement and extracapsular spread were significantly related to recurrence. Extracapsular spread and esophageal invasion had negative effects on survival. Such information can be used in patient counseling and appropriate risk stratification. In addition, these factors might be useful as markers to predict recurrence and prognosis of patients with T4a HPSCC.

## Abbreviations

CI: Confidence interval
DFS: Disease-free survival
DSS: Disease-specific survival
HPSCC: Hypopharyngeal squamous cell carcinoma
HR: Hazard ratio
